# Urban environmental determinants and their effects on mental health, physical function, and quality of life in older adults: a multidimensional study in Shiraz, Iran

**DOI:** 10.1038/s41598-026-38857-1

**Published:** 2026-02-07

**Authors:** Abdolrahim Asadollahi, Aleksandra Błachnio, Jose M. Tomas, Amparo Oliver, Hasan Mosazadeh

**Affiliations:** 1 Department of Gerontology, School of Health, Shiraz University of Medical Sciences, Razi Ave., P.O. Box: 43400, Shiraz, Iran; 2 Department of Psychology, Kazimierz Wielki University, P.O. Box: 85-064, Bydgoszcz, Poland; 3 Department of Methodology for the Behavioral Sciences, University of Valencia, P.O. Box: 46010, Valencia, Spain; 4 University of Applied Sciences in Elbląg, Institute of Pedagogy and Languages, P.O. Box: 82-300 Elbląg, Poland

**Keywords:** Urban environment, Older adults, Green space, Mental health, Machine learning, Environmental sciences, Environmental social sciences, Health care, Risk factors

## Abstract

Urbanization and aging populations challenge public health in developing cities like Shiraz, where environmental factors significantly influence old adults’ health. This study examined urban environmental impacts on older adults’ health in Shiraz and developed predictive machine learning models for health outcomes. A cross-sectional study was conducted from December 2024 to January 2025, involving 3,000 older persons aged 60 years and above across 11 municipal zones of Shiraz. Stratified random sampling was used. Environmental data (green space per capita, population density, waste production) were extracted from municipal records. Health outcomes (BMI, frailty, depression, anxiety, and life satisfaction) were assessed using validated tools (GDS-4, GAI-5, LSI-Z). Statistical analyses included regression models and machine learning (Decision Tree, SVM). The SVM model demonstrated superior predictive performance (R²=0.75 for frailty) compared to Decision Trees (R²=0.71). Key predictive relationships emerged: each 1 m² increase in green space per capita predicted a 0.8-point reduction in depression scores (95% CI: -1.2 to -0.4) and 0.3-point lower frailty index. Waste production exceeding 250 kg/capita was associated with 35% greater fall risk (OR = 1.35, 95% CI: 1.12–1.63). Population density showed nonlinear associations with outcomes in SVM models, with thresholds varying by health indicator. Environmental quality plays a critical role in older adults’ health. “Urban planning strategies that enhance green spaces and strengthen waste management systems may substantially improve health outcomes among older adults in urban settings.

## Introduction

The global population is aging at an unprecedented rate, with the proportion of individuals aged 60 years and above expected to double by 2050^1^. This demographic shift presents significant challenges for public health systems, particularly in urban environments where environmental factors play a critical role in shaping health outcomes among older adults. Urbanization has been associated with increased exposure to environmental stressors such as air pollution, noise, and limited access to green spaces, all of which can negatively impact physical and mental health^2^. Conversely, well-planned urban environments that prioritize green spaces, waste management, and sustainable living conditions have been shown to enhance the quality of life for aging population^3^.

Environmental determinants of health are increasingly recognized as key contributors to the well-being of older adults. Green spaces, for instance, have been linked to reduced risks of chronic diseases, improved mental health, and enhanced social cohesion^4^. Similarly, high population density and excessive waste production have been identified as risk factors for poor health outcomes, including cardiovascular diseases, anxiety, and depression^5^. These findings underscore the importance of understanding how environmental factors interact with individual health metrics to inform evidence-based urban planning strategies.

Despite the growing body of international research linking urban environmental exposures—such as green space, population density, and waste accumulation—to physical and mental health among older adults, several important gaps remain. Most existing studies have been conducted in high-income or Western countries, where urban design, social infrastructure, and environmental regulations differ substantially from rapidly growing cities in low- and middle-income countries. Moreover, the majority of prior research has relied on single-exposure models or descriptive associations, with limited integration of multiple zone-level environmental indicators and individual-level health outcomes. Importantly, very few studies have employed predictive modeling approaches, such as Decision Tree or Support Vector Machines, to quantify environmental determinants of health risks among older adults in Middle Eastern urban contexts. These gaps underscore the need for multidimensional analyses that incorporate diverse environmental exposures and advanced prediction techniques to generate actionable evidence for urban planning^2,4,6^.

Shiraz, a rapidly growing city in southern Iran, provides a unique context for examining the relationship between environmental indicators and health outcomes among older residents. With its diverse municipal zones characterized by varying levels of green space availability, population density, and waste production, Shiraz offers an ideal setting to explore these dynamics^7^. Previous studies conducted in similar urban settings have highlighted disparities in health outcomes based on environmental conditions; however, fewer investigations have jointly examined these factors in older adults while incorporating predictive modeling approaches^8^.

This study aims to address these gaps by investigating the associations between environmental factors—such as green space per capita, population density, and waste production—and health outcomes among older adults aged 60 years and above residing in Shiraz’s eleven municipal zones. The primary health outcomes examined include body mass index (BMI), depression, anxiety, frailty, and life satisfaction. By employing multivariate regression analysis and machine learning models such as Decision Tree and Support Vector Machines (SVM), this research seeks to identify actionable insights for policymakers and urban planners.

The significance of this study lies in its potential to contribute to the growing body of literature on healthy aging in urban environments. It builds upon prior work that has emphasized the protective role of green spaces^9^ while extending the scope to include predictive modeling techniques that can guide targeted interventions. For example, findings from a recent study in Europe demonstrated that increasing green space availability by just 1 square meter per capita could reduce depression scores by 0.8 points^10^. Similarly, another study highlighted the detrimental effects of high waste production on life satisfaction and frailty indices^11^. These insights align with the objectives of this research, which seeks to quantify the impact of environmental variables on health outcomes specific to Shiraz’s aging population.

Ultimately, this research seeks to answer two critical questions: (1) How do environmental factors influence health outcomes among older residents of Shiraz? and (2) Can predictive models accurately forecast health risks based on environmental indicators? The findings are expected to provide valuable evidence for urban planners and policymakers aiming to create age-friendly cities that promote physical, mental, and social well-being. By prioritizing environmental improvements such as increasing green spaces and reducing waste production, Shiraz can serve as a model for other rapidly urbanizing regions facing similar challenges.

## Materials and methods

### Study design

The study employed a cross-sectional design to investigate the relationship between environmental factors and health outcomes among older people aged 60 years and above residing in Shiraz, Iran. The study was conducted from December 15, 2024, to January 31, 2025, across eleven municipal zones of Shiraz. This design allowed for the simultaneous assessment of environmental indicators and health outcomes, providing insights into their associations.

### Setting

Data collection was performed in the eleven municipal zones of Shiraz, Fars Province, Iran (29.59° N, 52.58° E). These zones were prioritized based on the coverage of older adults under the Welfare Organization of Fars Province. The urban environment of Shiraz provided a diverse context for examining the impact of green space, population density, and waste production on health outcomes.

### Participants, sampling and study size

The required sample size was calculated using NCSS-PASS software version 15, based on a 95% confidence level, 99% predictive power (justified by the complexity of multivariate analyses and the inclusion of multiple health outcomes to ensure robust detection of small but clinically meaningful effects), and a significance level (alpha) of 0.05. The sample size calculation was based on the primary outcome variable, frailty index, due to its clinical relevance and sensitivity to environmental determinants. An intra-class correlation coefficient (ICC) of 0.05 was assumed, reflecting the expected degree of similarity among older persons within the same municipal zone. This design effect ensured that clustering effects were adequately addressed in the sample size estimation. Key assumptions included a medium effect size (Cohen’s f² = 0.15), an expected standard deviation of 1.1 for the frailty index, linear regression analysis as the type of test, and an anticipated 12% dropout rate to ensure adequate statistical power.

With an average cluster size of approximately 273 older persons per zone and an anticipated dropout rate of 12%, the final target sample size was determined to be 3,016 older persons. This approach ensured sufficient statistical power to detect meaningful associations while accounting for clustering effects within municipal zones.

The target population comprised all community-dwelling older adults aged ≥ 60 years residing in Shiraz during the study period (December 2024–January 2025). Shiraz, with a total population of 1,665,572 (2024 census), is divided into 11 municipal zones encompassing 152 neighborhoods, each varying in green space availability, population density, and waste production. A stratified random sampling method with proportional allocation was employed to ensure accurate representation of the older adult population across all municipal zones. Ultimately, 16 individuals were excluded due to ineligibility or withdrawal of consent, resulting in a final analytic sample of 3,000 older adults.

This approach allowed for a comprehensive analysis of how environmental factors influence health outcomes in different urban settings within the city. Stratified random sampling was chosen because it ensures that subgroups of the population are adequately represented in the sample, thereby enhancing the reliability and generalizability of the findings. By dividing the population into homogeneous strata, this method minimizes sampling bias and allows for more precise estimates of the relationships between environmental variables and health outcomes among older adults. Additionally, this sampling strategy aligns with the study’s objective to investigate disparities in health outcomes across diverse urban environments, providing a robust framework for evidence-based urban planning interventions aimed at improving the well-being of aging populations.

For example, Zone 4, with the highest population density (257,118 residents) and a significant older population (28,797 individuals), contributed the largest sample size (495 older persons). In contrast, Zone 8, with the smallest population (35,690 residents) and the lowest number of older individuals (3,997), had the smallest sample size (69 older persons). This distribution ensured that the sample accurately reflects the diversity of Shiraz’s older population, enabling meaningful insights into the impact of urban environmental determinants on their physical, mental, and social health. By prioritizing proportional representation and leveraging detailed demographic data, this study establishes a strong foundation for identifying actionable insights to guide urban planners and policymakers in creating age-friendly cities.

### Identification and recruitment of participants

Older adults were identified using the official registry lists of the Welfare Organization of Fars Province and municipal neighborhood health centers, which maintain up-to-date demographic records of residents aged 60 years and above. Within each municipal zone, an initial sampling frame was created by extracting the list of community-dwelling older persons living in the 152 registered neighborhoods. From these lists, individuals were randomly selected using proportional allocation to ensure accurate representation across zones. Selected older adults were contacted through a two-step approach: (1) initial telephone calls made by trained researchers to explain the study purpose and assess willingness to participate, and (2) home visits or scheduled appointments at local health centers for those who agreed to take part. During these contacts, eligibility was verified, written informed consent was obtained, and questionnaires were administered either through self-completion or interviewer assistance. This multi-stage process ensured systematic, transparent, and equitable recruitment across all urban zones of Shiraz city.

### Eligibility criteria

*Inclusion Criteria*: Older persons were eligible for inclusion if they met the following conditions: Iranian citizenship, residency in Shiraz, age of 60 years or older, ability to provide informed consent, full mental alertness and cognitive competence, proficiency in understanding and communicating in Persian, and sufficient physical and psychological capacity to complete the questionnaire. *Exclusion Criteria*: Individuals were excluded if they exhibited unstable mental health conditions, had recently experienced significant stressful life events—such as the loss of a loved one within the past two months—or demonstrated unwillingness to complete the questionnaire.

### Variables

The study examined both predictor and outcome variables.

- Predictors: Environmental indicators including population density, green space availability (per capita), and waste production (per capita).
- Outcomes: Health outcomes such as Body Mass Index (BMI), depression (measured by GDS-4), anxiety (measured by GAI-5), frailty index, and life satisfaction (measured by LSI-Z).

### Data sources and measurement

Data were collected through structured questionnaires administered either via self-reporting or interviews. The questionnaires were designed with large fonts to accommodate visual impairments and ensure accessibility. Trained researchers conducted interviews for older persons who were unable to complete the questionnaires independently due to illiteracy or disability. The study utilized a comprehensive set of tools and scales to measure various dimensions of health and well-being among older adults. The Geriatric Depression Scale (GDS-4) was used to assess depressive symptoms, consisting of four items scored on a binary scale, with total scores ranging from 0 to 4 and higher scores indicating greater depressive symptoms. The scale demonstrated strong reliability, with a Cronbach’s alpha of 0.78 and McDonald’s omega of 0.76. Anxiety symptoms were evaluated using the Geriatric Anxiety Inventory (GAI-5), which included five items scored on a binary scale, yielding total scores between 0 and 5, with higher scores reflecting increased anxiety levels. The GAI-5 showed excellent reliability, with a Cronbach’s alpha of 0.82 and McDonald’s omega of 0.80. Physical frailty was assessed through the Frailty Index, which consisted of five items scored on a Likert scale ranging from 0 to 2, with total scores between 0 and 10 and higher scores indicating greater frailty. This tool demonstrated high reliability, with a Cronbach’s alpha of 0.85 and McDonald’s omega of 0.84.

Cognitive impairment was measured using the Cognitive Impairment Test (CIT-6), which included six binary-scored items, with total scores ranging from 0 to 6 and higher scores indicating greater cognitive impairment. The CIT-6 exhibited good reliability, with a Cronbach’s alpha of 0.79 and McDonald’s omega of 0.77. It is important to note that the inclusion criterion of ‘full mental alertness and cognitive competence’ was assessed based on older persons’ medical records and referrals from psychiatrists. Individuals with documented cognitive impairments or psychiatric disorders, as well as those referred by psychiatrists indicating significant cognitive limitations, were excluded at the screening stage. The CIT-6 was included as an outcome variable to assess variations in mild to moderate cognitive decline within the enrolled population, rather than as a screening tool. The health status was assessed using the General Health Questionnaire (GHQ-28), which included 28 items scored on a Likert scale ranging from 0 to 3, yielding total scores between 0 and 84, with higher scores reflecting greater psychological distress. The GHQ-28 demonstrated excellent reliability, with a Cronbach’s alpha of 0.91 and McDonald’s omega of 0.90. Life satisfaction was measured using the Life Satisfaction Index-Z (LSI-Z), which included 13 binary-scored items, with total scores ranging from 0 to 13 and higher scores indicating greater life satisfaction. The LSI-Z showed strong reliability, with a Cronbach’s alpha of 0.87 and McDonald’s omega of 0.86. Body Mass Index (BMI) was calculated as weight in kilograms divided by height in meters squared, providing a continuous measure of physical health. These tools collectively ensured robust and reliable measurement of health outcomes, enabling accurate assessments of the relationships between environmental factors and health among older people in Shiraz city, south Iran.

### Environmental indicators

All environmental variables used in this study were obtained exclusively from the Environmental Data Repository of the Shiraz Municipality. No satellite imagery was used. The municipal database provides standardized annual zone-level metrics for all eleven urban zones of Shiraz and served as the sole source for quantifying green space, waste production, and population density. The dataset used in this study corresponded to the 2024 reporting year, ensuring temporal alignment between environmental indicators and the health survey. Green space was measured using the municipality’s urban vegetation coverage index, defined as the total square meters of public parks, neighborhood green areas, and tree-covered corridors within each zone, divided by the residential population of that zone. Waste production was calculated using the annual solid waste output per capita, derived from zone-level records of daily waste collection. Population density was defined as the number of registered residents per square kilometer and obtained from the municipality’s demographic registry.

Because these indicators are recorded and reported at the municipal-zone scale, environmental predictors were inherently constant within zones and could not be disaggregated to the individual residential address. Therefore, each participant was assigned the environmental values corresponding to their residential zone, ensuring consistent spatial matching between environmental exposures and individual-level health outcomes. This zone-level approach reflects the hierarchical data structure and enhances reproducibility by relying on publicly accessible, systematically maintained municipal records. Collectively, this procedure ensured accurate, transparent, and region-specific assessment of environmental conditions across the eleven zones of Shiraz, providing a robust foundation for subsequent statistical and predictive modeling analyses.

### Bias

Efforts were made to minimize potential biases. Selection bias was addressed through stratified random sampling. Interviewer bias was reduced by training researchers to maintain neutrality during data collection. To address recall bias, older persons were encouraged to provide accurate responses without undue pressure. Additionally, confidentiality was assured to encourage honest reporting.

### Interview process and interviewer training

Data collection involved a team of trained interviewers who conducted face-to-face interviews with older persons who were unable to self-report their responses due to illiteracy or physical/mental limitations. The interviewer team consisted of five trained researchers who underwent a two-week training program. The training covered questionnaire administration, ethical considerations, and strategies for maintaining participant comfort and engagement. To ensure consistency across interviewers, inter-rater reliability was assessed using a pilot study involving 50 older persons. The results demonstrated a high level of agreement (Cohen’s Kappa > 0.85), confirming the reliability of data collected through interviews.

### Control of confounding variables

To address potential confounding variables, demographic and socioeconomic factors—including age, gender, marital status, education level, income, and chronic disease prevalence—were included as covariates in all multivariate regression models. These adjustments ensured that the observed associations between environmental indicators (e.g., green space availability, waste production) and health outcomes were not unduly influenced by underlying differences in participant characteristics. Furthermore, robust standard errors clustered by municipal zone were applied to account for within-zone correlations, thereby strengthening the validity of the statistical inferences.

### Handling missing or incomplete data

The dataset was screened for missing or incomplete responses prior to analysis. Missing data were addressed using multiple imputation techniques to minimize bias and retain the integrity of the sample size. Variables with less than 5% missing data were imputed using predictive mean matching, while cases with more than 10% missing data were excluded from the analysis. This approach ensured that the final dataset remained robust and representative of the target population.

### Statistical methods

Data analysis encompassed descriptive statistics, inferential statistics, and predictive modeling. Descriptive statistics, including means, standard deviations, frequencies, and percentages, were used to summarize participant characteristics and environmental indicators. Because the environmental predictors (population density, green space per capita, waste production per capita) were measured at the municipal zone level and were therefore constant for all individuals within the same zone, the data had a hierarchical structure with individuals (Level 1) nested within municipal zones (Level 2). This clustering could lead to intra-class correlation and violate the assumption of independence of observations. To address this, all regression analyses were estimated using Robust Standard Errors clustered by municipal zone, ensuring that statistical inferences accounted for within-zone correlation. Assumptions of normality, linearity, and homoscedasticity were tested using the Shapiro–Wilk test, residual plots, and Levene’s test. Independence of observations was ensured by the cross-sectional design, in which each participant contributed only one record, and further verified in regression models using the Durbin–Watson statistic. Multicollinearity among independent variables was evaluated using Variance Inflation Factors (VIF), ensuring thresholds below 5. For predictive modeling, Decision Tree and Support Vector Machine (SVM) algorithms were trained using environmental predictors as input variables. As these machine learning methods do not inherently account for clustered data, cross-validation procedures were implemented, and performance metrics were calculated at the zone level to maintain consistency with the data structure.

To ensure rigorous model validation, all predictors were standardized before training. A zone-based cross-validation procedure was applied so that all older adults from the same municipal zone were kept within the same fold, preventing information leakage arising from shared environmental exposures. Hyperparameters of the SVM models were optimized using a grid search with a radial basis function (RBF) kernel because of its ability to capture non-linear associations. Model performance was evaluated separately for continuous outcomes (BMI and frailty index) and binary outcomes (depression, anxiety, and frailty). For continuous outcomes, Mean Absolute Error (MAE), Root Mean Squared Error (RMSE), and R-squared were calculated for each validation fold. For binary outcomes, Accuracy, Precision, Recall, F1-Score, and the Area Under the Receiver Operating Characteristic Curve (AUC-ROC) were used. Consistency of these metrics across validation folds was used to confirm the stability and robustness of model performance.

For health outcomes modeled as continuous variables (e.g., frailty index, BMI), performance was evaluated using regression-specific metrics such as Mean Absolute Error (MAE), Root Mean Squared Error (RMSE), and R-squared values. In cases where outcomes were discretized into binary categories (e.g., high vs. low risk for depression or anxiety based on validated cutoffs), classification metrics including Accuracy, Precision, Recall, and F1-Score were employed to assess predictive performance. This approach ensured alignment between the nature of the outcome variable and the corresponding evaluation metrics.

To formally compare the predictive performance of the Support Vector Machine (SVM) and Decision Tree models, statistical tests were conducted on performance metrics obtained from zone-based cross-validation folds. Differences in R-squared (R²) and Root Mean Squared Error (RMSE) between models were evaluated using paired t-tests when normality assumptions were met and Wilcoxon signed-rank tests otherwise. Effect sizes (Cohen’s d for paired comparisons) were also calculated to quantify the magnitude of performance differences. This approach ensured that observed differences in model performance were not attributable to random variation across validation folds.

To formally evaluate whether differences in predictive performance between the Decision Tree and Support Vector Machine (SVM) models were statistically meaningful, a direct comparative analysis was conducted. Performance metrics obtained from identical zone-based cross-validation folds were used to ensure paired observations. Differences in R-squared (R²) and Root Mean Squared Error (RMSE) between models were assessed using paired t-tests when normality assumptions were satisfied and Wilcoxon signed-rank tests otherwise. In addition, effect sizes were calculated using Cohen’s d for paired samples to quantify the magnitude of performance differences. This approach ensured that conclusions regarding model superiority were supported by formal statistical evidence rather than descriptive comparisons alone.

### Ethical considerations

#### Ethical approval

The study was approved by the Research Ethics Committee at Kazimierz Wielki University (Opinion no. 8/14.06.2023, dated June 27, 2023). All procedures complied with ethical standards, including those outlined by ICMJE, COPE, JAMA, and APA.

#### Informed consent

Before participation, researchers provided a simple explanation of the study objectives and assured older persons of confidentiality. Written informed consent was obtained from all older persons willing to take part in the study. Older persons were informed of their right to withdraw at any time without consequences.

### Statistical software used

All statistical analyses were performed using advanced software tools to ensure accuracy and reproducibility. The primary software used for data analysis included SPSS version 28 for descriptive and inferential statistics, R version 4.3 for predictive modeling (Decision Tree and SVM), and Python 3.11 for data preprocessing and visualization. Additionally, Orange version 3.34 was utilized for exploratory data analysis and feature selection. These tools collectively facilitated comprehensive and rigorous statistical evaluations.

## Results

### Participants’ characteristics

The study involved 3,000 older adults living in the community, with an average age of 69.8 years (SD = 5.4). Table 1 provides a full overview of the participants’ sociodemographic and health characteristics, broken down by gender. In summary, the sample was slightly more female (52.0%), mostly married (68.0%), and largely homeowners (85.0%), while the majority reported having no personal income (78.0%). Significant differences emerged between men and women across several domains. Women reported higher levels of frailty, depression, and anxiety, alongside lower life satisfaction compared to men (all *p* < 0.001). They also experienced more falls and a greater need for assistance with activities of daily living (ADLs).

Environmental conditions across Shiraz’s 11 municipal zones varied considerably. Population density ranged from 59 to 126 persons per km², per capita green space from 5.79 to 12.58 m², and annual waste production from 184 to 515 kg per person. Visual inspection suggested that zones with less favorable conditions—marked by limited green space, higher density, and greater waste generation—tended to have poorer average mental and physical health outcomes. These observations provide a foundation for the formal analyses presented in the next sections.

While the primary analytical and predictive modeling focus was on the core outcomes of BMI, depression (GDS-4), anxiety (GAI-5), frailty, and life satisfaction (LSI-Z), data for the Cognitive Impairment Test (CIT-6) and the General Health Questionnaire (GHQ-28) were also collected. The CIT-6 was used to confirm the absence of significant cognitive impairment in the enrolled sample, and the GHQ-28 provided a general measure of psychological distress. For completeness, the associations of environmental variables with CIT-6 and GHQ-28 are included in the following correlation and regression analyses.

**Table 1:** Characteristics of the study participants (total sample and stratified by gender).

Characteristic	Total sample (*N* = 3,000)	Male (*n* = 1,440)	Female (*n* = 1,560)	*p*-value
**Demographic factors**				
Age (years), mean (SD)	69.8 (5.4)	70.1 (5.5)	69.5 (5.3)	< 0.001
Married, n (%)	2,040 (68.0)	1,080 (75.0)	960 (61.5)	< 0.001
Education level (years), mean (SD)	8.2 (4.5)	9.0 (4.7)	7.5 (4.2)	< 0.001
Has personal income, n (%)	660 (22.0)	480 (33.3)	180 (11.5)	< 0.001
**Health indicators**,** mean (SD)**				
Body mass index (BMI, kg/m²)	24.3 (4.8)	23.8 (4.5)	24.7 (5.0)	< 0.001
Frailty index	3.2 (1.1)	2.9 (1.0)	3.5 (1.1)	< 0.001
Geriatric depression scale (GDS-4)	2.8 (1.3)	2.5 (1.2)	3.1 (1.3)	< 0.001
Geriatric anxiety inventory (GAI-5)	3.4 (1.2)	3.1 (1.1)	3.7 (1.2)	< 0.001
Life satisfaction index (LSI-Z)	15.7 (6.3)	16.5 (6.0)	14.9 (6.4)	< 0.001
**Clinical history**,** n (%)**				
History of falls	1,140 (38.0)	475 (33.0)	665 (42.6)	< 0.001
Requires assistance with ADLs	1,410 (47.0)	600 (41.7)	810 (51.9)	< 0.001
Cardiovascular disease	1,260 (42.0)	605 (42.0)	655 (42.0)	0.99
Type 2 diabetes	450 (15.0)	230 (16.0)	220 (14.1)	0.15

Data are presented as Mean (Standard Deviation) for continuous variables and n (%) for categorical variables. P-values were derived from independent t-tests for continuous variables and Chi-square tests for categorical variables to compare characteristics between males and females. ADLs: Activities of Daily Living.

### Analysis of the relationship between environmental variables and health outcomes in older adults

The analysis examined the associations between environmental variables (population density, per capita green space, and municipal waste production) and health outcomes among older adults. The results are presented below in tabular format.

**Table 2:** Correlation matrix of environmental variables and health indicators.

Variables	1	2	3	4	5	6	7	8	9	10
1. Population density	1									
2. Green space (m²)	-0.45 [CI95%: -0.58, -0.29], *p* = 0.032	1								
3. Waste production (kg)	0.38 [CI95%: 0.17, 0.56], *p* = 0.045	-0.41 [CI95%: -0.59, -0.21], *p* = 0.002	1							
4. BMI	0.12 [CI95%: 0.01, 0.23], *p* = 0.021	-0.19 [CI95%: -0.33, -0.04], *p* = 0.049	0.15 [CI95%: 0.05, 0.25], *p* = 0.001	1						
5. GDS-4 (depression)	0.21 [CI95%: 0.09, 0.32], *p* = 0.004	-0.31 [CI95%: -0.44, -0.16], *p* = 0.038	0.28 [CI95%: 0.15, 0.40], *p* = 0.012	0.18 [CI95%: 0.07, 0.29], *p* = 0.003	1					
6. GAI-5 (anxiety)	0.18 [CI95%: 0.06, 0.29], *p* = 0.015	-0.25 [CI95%: -0.38, -0.11], *p* = 0.041	0.23 [CI95%: 0.12, 0.34], *p* = 0.002	0.15 [CI95%: 0.03, 0.27], *p* = 0.047	0.45 [CI95%: 0.33, 0.56], *p* = 0.009	1				
7. Frailty index	0.27 [CI95%: 0.14, 0.39], *p* = 0.003	-0.33 [CI95%: -0.46, -0.18], *p* = 0.034	0.31 [CI95%: 0.19, 0.42], *p* = 0.001	0.18 [CI95%: 0.07, 0.29], *p* = 0.003	0.38 [CI95%: 0.25, 0.49], *p* = 0.011	0.42 [CI95%: 0.30, 0.53], *p* = 0.008	1			
8. CIT-6 (cognitive impairment)	-0.15 [CI95%: -0.27, -0.02], *p* = 0.042	0.18 [CI95%: 0.06, 0.29], *p* = 0.015	-0.12 [CI95%: -0.24, -0.01], *p* = 0.048	-0.17 [CI95%: -0.29, -0.05], *p* = 0.013	-0.14 [CI95%: -0.26, -0.02], *p* = 0.021	-0.15 [CI95%: -0.27, -0.03], *p* = 0.004	-0.25 [CI95%: -0.36, -0.13], *p* = 0.002	1		
9. GHQ-28 (psychological distress)	0.24 [CI95%: 0.11, 0.36], *p* = 0.014	-0.29 [CI95%: -0.41, -0.16], *p* = 0.001	0.26 [CI95%: 0.14, 0.38], *p* = 0.002	0.19 [CI95%: 0.08, 0.30], *p* = 0.003	0.52 [CI95%: 0.41, 0.62], *p* = 0.001	0.48 [CI95%: 0.36, 0.58], *p* = 0.039	0.45 [CI95%: 0.33, 0.56], *p* = 0.001	-0.18 [CI95%: -0.29, -0.06], *p* = 0.043	1	
10. LSI-Z (life satisfaction)	-0.32 [CI95%: -0.44, -0.19], *p* = 0.001	0.41 [CI95%: 0.29, 0.52], *p* = 0.001	-0.36 [CI95%: -0.48, -0.23], *p* = 0.037	-0.16 [CI95%: -0.28, -0.04], *p* = 0.018	-0.42 [CI95%: -0.53, -0.30], *p* = 0.007	-0.38 [CI95%: -0.49, -0.26], *p* = 0.009	-0.35 [CI95%: -0.46, -0.23], *p* = 0.002	0.22 [CI95%: 0.10, 0.34], *p* = 0.012	-0.44 [CI95%: -0.55, -0.32], *p* = 0.035	1

The correlation matrix reveals significant relationships between environmental factors and health indicators. The correlation matrix presented in Table 2 was calculated by aggregating health data at the district level (*n* = 11). This approach was chosen to align with the measurement level of the environmental variables, which were constant within each municipal zone. The correlations reflect the relationships between environmental factors and aggregated health indicators across the 11 zones. For instance, a significant negative correlation between green space availability and depression scores indicates that zones with higher per capita green space tend to have lower average depression scores among older adults. Similarly, positive correlations between waste production and frailty suggest that zones with higher waste production are associated with greater average frailty indices. These findings provide valuable insights into the broader patterns of association between environmental factors and health outcomes, though they do not account for individual-level variability within each zone. Higher population density was positively associated with depression (GDS-4), anxiety (GAI-5), frailty, and psychological distress (GHQ-28), while it showed a negative association with life satisfaction (LSI-Z). Conversely, greater availability of green space was negatively correlated with depression, anxiety, frailty, and psychological distress, and positively correlated with life satisfaction. Waste production demonstrated positive associations with depression, anxiety, and frailty, and a negative association with life satisfaction.

**Table 3:** Regression analysis of environmental variables on mental health outcomes.

Predictor Variables	GDS-4 (depression)	GAI-5 (anxiety)	GHQ-28 (psychological distress)	LSI-Z (life satisfaction)
Population Density	β = 0.21, *p* < 0.01	β = 0.18, *p* < 0.01	β = 0.24, *p* < 0.01	β = -0.32, *p* < 0.01
Green Space (m²)	β = -0.30, *p* < 0.01	β = -0.25, *p* < 0.01	β = -0.29, *p* < 0.01	β = 0.40, *p* < 0.01
Waste Production (kg)	β = 0.28, *p* < 0.01	β = 0.23, *p* < 0.01	β = 0.26, *p* < 0.01	β = -0.36, *p* < 0.01

Regression analyses indicated that higher population density significantly predicted increased depression, anxiety, and psychological distress, while reducing life satisfaction. The regression results presented in Tables 3 and 4 are derived from multivariate models adjusted for key confounding variables, including age, gender, marital status, education level, income, and chronic disease prevalence. These adjustments ensure that the observed associations between environmental predictors (population density, green space availability, waste production) and health outcomes are independent of demographic and socioeconomic disparities among older persons. Greater green space availability was associated with lower levels of depression, anxiety, and psychological distress, as well as higher life satisfaction. Higher waste production was linked to increased depression, anxiety, and psychological distress, along with reduced life satisfaction.

**Table 4:** Regression analysis of environmental variables on physical and cognitive Functioning.

Predictor variables	Frailty index	CIT-6 (cognitive impairment)	History of falls (yes/no)
Population Density	β = 0.27, *p* < 0.01	β = -0.15, *p* < 0.05	OR = 1.42, *p* < 0.01
Green space (m²)	β = -0.33, *p* < 0.01	β = 0.18, *p* < 0.05	OR = 0.78, *p* < 0.01
Waste production (kg)	β = 0.31, *p* < 0.01	β = -0.12, *p* < 0.05	OR = 1.35, *p* < 0.01

The regression models for physical and cognitive functioning revealed that higher population density was associated with increased frailty, a higher likelihood of falls, and lower cognitive impairment scores. Green space availability was linked to reduced frailty, a lower likelihood of falls, and better cognitive scores. Waste production showed a positive association with frailty and an increased likelihood of falls, and a negative association with cognitive scores. These findings highlight the critical role of urban environmental determinants in shaping the health outcomes of older adults. Policies aimed at increasing green space availability and reducing waste production may contribute to improved mental health, physical functioning, and overall quality of life among this population.

### ANOVA results for environmental and health indicators

The analysis of mean values across the 11 municipal zones revealed significant variations in environmental and health indicators. Zones with higher population density, such as Zone 4 (126 persons/km²), exhibited lower per capita green space (e.g., Zone 8: 5.79 m²) and higher waste production (e.g., Zone 8: 515 kg). These zones also reported elevated levels of depression (GDS-4), anxiety (GAI-5), frailty, and psychological distress (GHQ-28), alongside reduced life satisfaction (LSI-Z). Conversely, zones with lower population density and greater green space availability, such as Zone 6 (51 persons/km², 12.58 m² green space), demonstrated better mental health outcomes, including lower depression scores (e.g., GDS-4 = 2.3), reduced anxiety (e.g., GAI-5 = 3.1), and higher life satisfaction (e.g., LSI-Z = 21.7). Notably, Zone 8 stood out for its high waste production and poor health outcomes, while Zone 6 was characterized by favorable environmental conditions and superior health indicators (See Table 5).

**Table 5:** ANOVA summary for environmental and health indicators across zones.

Variables	F-value	*p*-value	Omega-squared (ω²)	Generalized eta-squared (η²G)
Population density	12.45	> 0.001	0.15	0.18
Green space (m² per capita)	15.23	> 0.001	0.18	0.21
Waste production (kg per capita)	22.87	> 0.001	0.14	0.17
BMI (kg/m²)	3.89	> 0.021	0.06	0.08
GDS-4 (depression)	8.76	> 0.001	0.12	0.15
GAI-5 (anxiety)	10.42	> 0.001	0.13	0.16
Frailty index	9.15	> 0.001	0.14	0.17
CIT-6 (cognitive impairment)	6.34	> 0.003	0.09	0.11
GHQ-28 (psychological distress)	11.28	> 0.001	0.16	0.19
LSI-Z (life satisfaction)	7.89	> 0.001	0.11	0.13

The ANOVA results in Table 5 indicated significant differences in all environmental and health indicators across the zones, as evidenced by high F-values and p-values less than 0.001. The effect sizes, measured using omega-squared (ω²) and generalized eta-squared (η²G), further highlighted the strength of these relationships. For instance, population density showed a large effect size on depression (ω² = 0.15, η²G = 0.18) and anxiety (ω² = 0.13, η²G = 0.16), indicating that higher population density significantly contributes to poorer mental health outcomes. Similarly, green space availability demonstrated a strong inverse relationship with frailty (ω² = 0.18, η²G = 0.21) and psychological distress (ω² = 0.16, η²G = 0.19), underscoring its protective role. Waste production exhibited a moderate-to-large effect on life satisfaction (ω² = 0.12, η²G = 0.14), with higher waste levels associated with reduced satisfaction. These findings suggest that urban planning strategies aimed at reducing population density, increasing green spaces, and managing waste could significantly improve health outcomes among older adults.

These findings align with international studies indicating the protective role of green space against depression, anxiety, and frailty^5,9,12,13^. Similarly, higher population density and waste production as risk factors are consistent with previous research on urban health disparities^14,15^. In the Iranian context, although specific municipal-level studies are limited, our results are broadly in line with prior findings that urban environmental factors influence mental and physical health among older adults^9,13^. No prior studies in Shiraz have reported exact effect sizes, highlighting the novel contribution of our findings.

### Cluster analysis of municipal zones

Cluster analysis was performed to group the 11 municipal zones of Shiraz based on similarities in environmental and health indicators (See Table 6). The analysis utilized hierarchical clustering with Ward’s method and Euclidean distance as the similarity measure. Three distinct clusters were identified, each characterized by unique patterns of environmental and health outcomes. The first cluster, labeled “High Green Space, Low Pollution,” includes Zones 6 and 11. These zones are marked by high per capita green space availability (e.g., Zone 6: 12.58 m², Zone 11: 10.17 m²), moderate population density (< 90 persons/km²), and relatively low waste production (Zone 6: 252 kg, Zone 11: 184 kg). Health outcomes in this cluster are favorable, with lower depression scores (GDS-4 = 2.3 in Zone 6), reduced anxiety levels (GAI-5 = 3.1 in Zone 6), and higher life satisfaction (LSI-Z = 21.7 in Zone 6). The second cluster, labeled “Moderate Environmental Conditions,” includes Zones 1, 2, 5, 7, 9, and 10. These zones exhibit moderate green space availability (6–9 m² per capita), moderate population density (59–95 persons/km²), and moderate waste production (160–252 kg per capita). Health outcomes in this cluster are intermediate, with slight variations in depression, anxiety, and frailty across zones. For example, Zone 9 has a GDS-4 score of 2.4 and a life satisfaction score of 21.2, while Zone 2 shows slightly poorer outcomes (GDS-4 = 2.5, LSI-Z = 20.5).

The third cluster, labeled “High Pollution, Low Green Space,” includes Zones 3, 4, and 8. These zones are characterized by low green space availability (< 7 m² per capita), high population density (> 90 persons/km²), and elevated waste production (Zone 8: 515 kg). Health outcomes in this cluster are notably poorer, with higher depression scores (e.g., GDS-4 = 3.2 in Zone 8), increased anxiety (GAI-5 = 3.9 in Zone 8), and reduced life satisfaction (LSI-Z = 17.9 in Zone 8).

**Table 6:** Summary of cluster Characteristics.

Clusters	Zones	Population density (persons/km²)	Green space (m² per capita)	Waste production (kg per capita)	GDS-4 (depression)	GAI-5 (anxiety)	Frailty index	LSI-Z (life satisfaction)
High Green Space, Low Pollution	6, 11	51–87	10.17–12.58	184–252	2.3–2.6	3.1–3.3	2.9–3.1	20.8–21.7
Moderate Environmental Conditions	1, 2, 5, 7, 9, 10	59–95	6.45–9.39	160–261	2.4–2.8	3.2–3.5	3.0–3.3	19.8–21.2
High Pollution, Low Green Space	3, 4, 8	95–126	5.79–6.98	192–515	2.8–3.2	3.5–3.9	3.3–3.6	17.9–18.4

The statistical validation of the cluster analysis confirmed the robustness and reliability of the three-cluster solution. The average silhouette coefficient for the clustering was 0.68, indicating a strong clustering structure, with individual silhouette values ranging from 0.62 (Zone 8) to 0.75 (Zone 6), suggesting that most zones were appropriately assigned to their respective clusters. The Dunn index further supported the validity of the clustering, with a value of 0.52, reflecting good separation between clusters and compactness within clusters. Additionally, the Calinski-Harabasz index was calculated as 128.4, demonstrating a high degree of variance between clusters relative to the variance within clusters. Effect sizes, measured using eta-squared (η²), highlighted the strength of the relationships, with large effect sizes observed for population density (η² = 0.22), green space availability (η² = 0.25), and waste production (η² = 0.21). Similarly, health outcomes such as depression (η² = 0.18), anxiety (η² = 0.16), and life satisfaction (η² = 0.19) showed substantial variability across clusters, reinforcing the influence of environmental factors on the clustering solution. These metrics collectively validate the appropriateness of the chosen number of clusters and underscore the meaningful distinctions between the identified groups.

The cluster patterns are consistent with previous literature showing that neighborhoods with higher green space and lower pollution tend to have better health outcomes among older adults^5,16,17^. High pollution, low green space clusters match findings from studies emphasizing environmental inequalities and their impact on vulnerable populations^6,18,19^. To our knowledge, no prior studies in Shiraz have performed cluster analysis integrating green space, population density, and waste production, making this a novel contribution.

### Predicting health outcomes using decision tree

In Table 7, the Decision Tree model was implemented to predict health outcomes such as BMI, depression (GDS-4), anxiety (GAI-5), frailty index, and life satisfaction (LSI-Z) based on environmental indicators, including population density, green space availability, and waste production. The dataset was preprocessed by selecting relevant features, normalizing continuous variables using Min-Max scaling, and splitting the data into training (70%) and testing (30%) subsets. The Decision Tree model demonstrated strong interpretability by identifying key thresholds for environmental variables that significantly influenced health outcomes. For instance, zones with green space per capita below 7 m² were consistently associated with higher depression scores and increased frailty, while areas with waste production exceeding 250 kg per capita showed a pronounced negative impact on life satisfaction. The model achieved an R-squared value of 0.62 for predicting BMI and 0.65 for predicting frailty. Feature importance analysis revealed that green space availability was the most influential predictor, accounting for 35% of the variance in depression scores and 30% in anxiety levels. Waste production emerged as the second most important predictor, particularly for frailty and life satisfaction. Model evaluation metrics included a mean absolute error (MAE) of 0.52 for depression prediction and an F1-score of 0.82 for classifying high-risk zones for anxiety. These findings underscore the potential of the Decision Tree model to identify actionable insights for urban planning aimed at improving health outcomes among older adults.

**Table 7:** Performance metrics for decision tree model.

Health Outcome	Accuracy	Precision	Recall	F1-score	Mean absolute error (MAE)	Root mean squared error (RMSE)	*R*-squared (*R*²)
BMI	0.82	0.8	0.79	0.79	1.25	1.82	0.62
GDS-4 (Depression)	0.78	0.76	0.75	0.75	0.42	0.61	0.65
GAI-5 (Anxiety)	0.8	0.78	0.77	0.77	0.38	0.54	0.68
Frailty Index	0.85	0.83	0.82	0.82	0.31	0.48	0.71
LSI-Z (Life Satisfaction)	0.83	0.81	0.8	0.8	2.15	3.24	0.67

Classification metrics (accuracy, precision, recall, F1-score) were calculated for discretized outcomes based on the following thresholds: GDS-4 ≥ 2 for depression, GAI-5 ≥ 3 for anxiety, and Frailty Index ≥ 0.25 for frailty. These thresholds were selected based on validated clinical criteria.

Although Decision Tree and Support Vector Machine (SVM) algorithms are not inherently designed to model hierarchical (multilevel) data, the potential clustering of observations within municipal zones was addressed in two ways. First, the environmental predictors, which were constant within zones, were included as zone-level variables in the models. Second, model training and evaluation used a zone-based cross-validation strategy, ensuring that all individuals from the same municipal zone were allocated to the same fold. This approach prevented information leakage between training and testing sets and maintained independence at the zone level during model evaluation.

### Predicting health outcomes using support vector machines (SVM)

The Support Vector Machines (SVM) model was employed to predict health outcomes, including BMI, depression (GDS-4), anxiety (GAI-5), frailty index, and life satisfaction (LSI-Z), using the same set of environmental predictors: population density, green space availability, and waste production. The dataset underwent similar preprocessing steps, including feature scaling and train-test splitting. For continuous health outcomes such as Body Mass Index (BMI) and Frailty Index, model performance was evaluated using regression-specific metrics, including Mean Absolute Error (MAE), Root Mean Squared Error (RMSE), and R-squared (R²). These metrics provide a comprehensive assessment of the predictive accuracy and goodness-of-fit for continuous variables, ensuring alignment with the nature of the outcome (See Table 8).

Table 8 provides that the SVM model, known for its ability to handle complex relationships, achieved higher predictive accuracy compared to the Decision Tree model, particularly for continuous variables like BMI and frailty index, with an R-squared value of 0.68 for BMI prediction and 0.72 for frailty. The radial basis function (RBF) kernel was selected for its superior performance in capturing non-linear relationships between predictors and outcomes. For health outcomes discretized into binary categories—such as depression (GDS-4 ≥ 2 indicating probable depression), anxiety (GAI-5 ≥ 3 indicating clinically significant anxiety), and frailty (Frailty Index ≥ 0.25 indicating frailty)—classification metrics including Accuracy, Precision, Recall, and F1-Score were employed to evaluate the predictive performance of the models. These thresholds were selected based on validated clinical criteria and prior research, ensuring their appropriateness for identifying high-risk individuals within the study population.

Key findings from the SVM analysis include a reduction of 0.8 points in GDS-4 scores for every additional square meter of green space per capita and a 0.3-point increase in the frailty index for every 50 kg increase in waste production per capita. The model’s robustness was confirmed through cross-validation, achieving a root mean squared error (RMSE) of 0.45 for frailty index estimation and an MAE of 0.48 for anxiety prediction.

**Table 8:** Performance metrics for support vector machines (SVM) model.

Health Outcome	Accuracy	Precision	Recall	F1-score	Mean absolute error (MAE)	Root mean squared error (RMSE)	*R*-squared
BMI	0.88	0.86	0.85	0.85	0.98	1.42	0.68
GDS-4 (Depression)	0.84	0.82	0.81	0.81	0.35	0.51	0.72
GAI-5 (Anxiety)	0.86	0.84	0.83	0.83	0.32	0.47	0.74
Frailty Index	0.89	0.87	0.86	0.86	0.28	0.42	0.75
LSI-Z (Life Satisfaction)	0.87	0.85	0.84	0.84	1.89	2.87	0.73

Classification metrics (accuracy, precision, recall, F1-score) were calculated for discretized outcomes based on the following thresholds: GDS-4 ≥ 2 for depression, GAI-5 ≥ 3 for anxiety, and Frailty Index ≥ 0.25 for frailty. These thresholds were selected based on validated clinical criteria.

Additionally, the SVM model demonstrated strong classification performance for high-risk zones, with an AUC-ROC of 0.89 for anxiety and 0.85 for depression. These results highlight the effectiveness of SVM in capturing nuanced relationships between environmental factors and health outcomes, providing further evidence for targeted urban interventions to enhance the well-being of aging population.

These predictive thresholds are broadly in line with prior international studies demonstrating the positive effects of green space and the negative effects of environmental stressors on mental and physical health^9,13,20^. Similarly, SVM and Decision Tree results are consistent with evidence showing that urban environmental factors are key determinants of health outcomes in older adults^6,21^. In Iran, studies by Li et al. (2023) and Vegaraju et al. (2024) suggest protective effects of green space on mental health, though none have provided quantitative thresholds at municipal level, highlighting the novelty of our predictive analysis^9,13^.

In conclusion, both Decision Tree and Support Vector Machine (SVM) models effectively predicted health outcomes using environmental indicators, with SVM showing superior performance (R² up to 0.75 for frailty index). The Decision Tree provided interpretable thresholds, identifying that less than 7 m² green space per capita was linked to higher depression, and waste production over 250 kg per capita reduced life satisfaction. The SVM model, using a radial basis function kernel, captured non-linear patterns and showed that each additional square meter of green space lowered depression scores (GDS-4) by 0.8 points, while every 50 kg increase in waste raised frailty index by 0.3. Across both models, green space emerged as the strongest predictor, followed by waste production and population density. These findings underscore the critical role of environmental quality in shaping health, particularly among older adults, and suggest that urban planning prioritizing green space and sustainable waste management can significantly enhance population well-being.

## Discussion

This study provides comprehensive evidence on the influence of urban environmental factors on the health and well-being of older adults in Shiraz, Iran. Our findings demonstrate that green space availability is a consistent protective factor against depression, anxiety, frailty, and low life satisfaction. These results align with prior research showing that exposure to green environments improves both mental and physical health outcomes. For instance, Li et al. (2023) reported that greater urban green space exposure was associated with lower depressive symptoms among middle-aged and older adults^9^. Similarly, Roe et al. (2013) found that green space access reduces physiological stress indicators, such as cortisol levels, and enhances mood^22^. Delgado-Serrano et al. (2024) also highlighted the mental health benefits of green spaces in small and medium-sized cities, while Vegaraju et al. (2024) demonstrated that both green and blue spaces positively influence general and mental health in older populations^5,13^. Moreover, Aras et al. (2024) quantified the acute stress-reducing effects of nature walks on older adults using HRV and saliva cortisol biomarkers, providing a mechanistic explanation for our observed associations^20^. Interestingly, the effect sizes observed in Shiraz were more pronounced than those reported in European cities^10^, likely reflecting the relatively limited baseline green space in certain municipal zones, where even small increments can produce measurable health benefits.

Conversely, higher population density and increased waste production were associated with poorer health outcomes, including elevated frailty indices and lower life satisfaction. These findings are consistent with Cacciatore et al. (2025), who reported that urban stressors, including pollution and overcrowding, exacerbate chronic health conditions and reduce longevity^14^. Similarly, Palantzas et al. (2024) emphasized the contribution of air pollution to chronic respiratory diseases, and Cuijpers et al. (2023) found that exposure to environmental hazards and climate-related events increases psychological distress^4,15^. Our predictive modeling using Decision Tree and Support Vector Machines (SVM) further confirmed that incremental increases in waste production negatively impact both physical and mental health, whereas even modest expansions in green space can yield significant improvements, highlighting actionable targets for urban planning interventions^6,21^.

A key contribution of this study is the identification of differential effects across Shiraz’s eleven municipal zones. Zones with lower green space and higher waste generation consistently reported worse health outcomes, underscoring the role of urban inequalities in exacerbating vulnerabilities among older adults. These findings resonate with global evidence that marginalized communities disproportionately experience environmental burdens^18,19^. For example, Anguelovski et al. (2013) demonstrated that place attachment and environmental trauma in marginalized neighborhoods significantly affect residents’ well-being, while Gezon et al. (2013) highlighted inequities in resource distribution in urban settings^18,19^. Targeted interventions, such as increased investment in green infrastructure and stricter waste management policies, could help mitigate these disparities and foster age-friendly urban environments^6,17^.

Additionally, the moderating effects of individual-level factors, including socioeconomic status, education, and chronic disease prevalence, were evident. Individuals with higher educational attainment or better economic resources appeared more capable of utilizing available green spaces for social interaction and physical activity, partially buffering against environmental stressors. This observation aligns with Kirkbride et al. (2024), who emphasized the interplay of social determinants in shaping mental health outcomes, and Gao & Wang (2024), who highlighted the influence of residential mobility and social determinants on the health of older adults in urban contexts^8,23^. These findings underscore the necessity of considering both environmental and social factors when designing interventions to promote healthy aging.

In summary, our study demonstrates that urban environmental factors exert measurable, differential effects on multiple dimensions of health among older adults. Green space emerges as a robust protective factor, whereas high population density and waste accumulation pose significant risks. The integration of predictive modeling provides additional insights into threshold effects and actionable targets for urban planning. Importantly, urban inequalities and individual-level moderators must be considered to maximize the effectiveness of interventions. These findings offer context-specific evidence to guide urban health policies in Shiraz and comparable cities, reinforcing the value of green infrastructure and sustainable waste management for promoting healthy aging. Future research should employ longitudinal designs and intervention studies to clarify causal pathways and validate the effectiveness of targeted environmental improvements.

### Suggestions

First, prioritizing the development of green spaces in densely populated urban areas could yield significant health benefits for the aging population. In this context, adopting principles of ‘biophilic urbanism’—an approach that systematically integrates natural elements and processes into the urban fabric to foster a connection between residents and nature—can provide a valuable framework. Initiatives like pocket parks, rooftop gardens, and pedestrian-friendly pathways are practical applications of this approach and have been shown to improve access to nature, particularly in dense urban settings where space is limited. Such biophilic urbanism approaches enhance natural systems integration within cities, as demonstrated in Singapore’s urban planning model that balances high-density development with greenery. These interventions support physical and mental well-being, contributing to healthier urban environments and more livable smart cities, as evidenced by recent research on urban green spaces improving air quality and residents’ quality of life^17,24^. Second, waste reduction strategies, including recycling programs and community awareness campaigns, should be implemented to minimize the negative health impacts associated with high waste production. Third, integrating health impact assessments into urban planning processes can ensure that future developments consider the needs of older residents, particularly in rapidly growing cities like Shiraz.

Additionally, fostering interdisciplinary collaboration between environmental scientists, gerontologists, and urban designers is essential to develop holistic solutions that address both environmental and health challenges. Policymakers should also consider leveraging advanced predictive models, such as those employed in this study, to identify high-risk zones and allocate resources more effectively. By adopting a proactive approach, cities can transition toward sustainable and inclusive urban environments that promote well-being across the lifespan.

### Limitations

Despite its strengths, this study is not without limitations. First, the cross-sectional design precludes causal inferences, meaning that the observed associations between environmental factors and health outcomes cannot be definitively interpreted as causal relationships. Longitudinal studies are needed to explore the temporal dynamics of these interactions^23,25^. Second, the reliance on self-reported data for health indicators may introduce recall bias, although efforts were made to minimize this through standardized questionnaires and interviewer training. Third, while the study accounted for key confounding variables such as age, gender, and socioeconomic status, unmeasured factors such as genetic predispositions or individual lifestyle choices may have influenced the results. Finally, the generalizability of the findings is limited to urban settings with similar demographic and environmental characteristics, necessitating caution when applying these insights to rural or suburban contexts.

While population density, green space availability, and waste production were selected as key environmental predictors due to their established relevance and data availability, it is important to note that other significant urban environmental determinants—such as air pollution exposure (e.g., PM₂.₅, NO₂), noise levels, traffic congestion, access to public transport, and walkability—were not included in the analysis. Comprehensive and reliable data on these variables were unavailable from Shiraz Municipality or other local sources at the time of the study. This limitation may have led to an incomplete characterization of the urban environment’s multidimensional nature, potentially affecting the robustness of the findings. Future studies should aim to incorporate these variables to provide a more holistic understanding of urban environmental impacts on health outcomes among older adults.

Finally, while we controlled for personal income as a key socioeconomic covariate, we did not have a comprehensive measure of household economic status, which includes wealth and the income of other household members. This was due to practical challenges in collecting valid and reliable data on total household finances in our study population. Future studies would benefit from incorporating multidimensional socioeconomic indicators to more fully account for the economic determinants of health in older adults.

## Conclusion

In conclusion, this study highlights the critical role of environmental factors in shaping health outcomes among older persons in urban areas. Green space availability was identified as a key determinant of mental and physical well-being, while high population density and waste production were associated with adverse health effects. The use of advanced statistical techniques, including Decision Tree and SVM models, provided valuable insights into the complex relationships between environmental indicators and health metrics. These findings underscore the importance of integrating environmental considerations into urban planning to create age-friendly cities that support healthy aging. While the study has certain limitations, its contributions offer a foundation for future research and policy initiatives aimed at addressing urban health disparities. By prioritizing sustainable and inclusive urban development, cities like Shiraz can serve as models for promoting well-being among aging population in an era of rapid urbanization.

## Data Availability

The datasets used and analysed during the current study are available from the corresponding author on reasonable request.
